# Who Would Pay for State Alcohol Tax Increases in the United States?

**DOI:** 10.5888/pcd13.150450

**Published:** 2016-05-19

**Authors:** Timothy S. Naimi, James I. Daley, Ziming Xuan, Jason G. Blanchette, Frank J. Chaloupka, David H. Jernigan

**Affiliations:** Author Affiliations: James I. Daley, Boston University School of Medicine, Boston, Massachusetts; Ziming Xuan, Department of Community Health Sciences, Boston University School of Public Health, Boston, Massachusetts; Jason G. Blanchette, Boston Medical Center, Boston, Massachusetts; Frank J. Chaloupka, University of Illinois at Chicago, Chicago, Illinois; David H. Jernigan, Johns Hopkins Bloomberg School of Public Health, Baltimore, Maryland.

## Abstract

**Introduction:**

Despite strong evidence that increasing alcohol taxes reduces alcohol-related harm, state alcohol taxes have declined in real terms during the past 3 decades. Opponents of tax increases argue that they are unfair to “responsible” drinkers and those who are financially disadvantaged. The objectives of this study were to assess the impact of hypothetical state alcohol tax increases on the cost of alcohol for adults in the United States on the basis of alcohol consumption and sociodemographic characteristics.

**Methods:**

The increased net cost of alcohol (ie, product plus tax) from a series of hypothetical state alcohol tax increases was modeled for all 50 states using data from the 2011 Behavioral Risk Factor Surveillance System, IMPACT Databank, and the Alcohol Policy Information System. Costs were assessed by drinking pattern (excessive vs nonexcessive) and by sociodemographic characteristics.

**Results:**

Among states, excessive drinkers would pay 4.8 to 6.8 times as much as nonexcessive drinkers on a per capita basis and would pay at least 72% of aggregate costs. For nonexcessive drinkers, the annual cost from even the largest hypothetical tax increase ($0.25 per drink) would average less than $10.00. Drinkers with higher household incomes and non-Hispanic white drinkers would pay higher per capita costs than people with lower incomes and racial/ethnic minorities.

**Conclusion:**

State-specific tax increases would cost more for excessive drinkers, those with higher incomes, and non-Hispanic whites. Costs to nonexcessive drinkers would be modest. Findings are relevant to developing evidence-based public health practice for a leading preventable cause of death.

## Introduction

Excessive alcohol consumption is the fourth leading preventable cause of death in the United States, where it is responsible for approximately 88,000 deaths annually, nearly 70% of which involve adults aged 20 to 64 years (1,2). Excessive alcohol consumption is also a risk factor for unintentional injuries, violence, liver disease, stroke, dementia, hypertension, several types of cancer, sexual assault, fetal alcohol spectrum disorders, and alcohol use disorders (3,4). In addition, excessive alcohol use cost the United States $249 billion in 2010, or approximately $2.05 per drink, and about $2.00 of every $5.00 were paid by government (5).

Although alcohol taxes have been used to raise revenue, part of their historical justification has also been their public health benefit (6). Among alcohol policy interventions, evidence of effectiveness in reducing excessive drinking and related harms is the strongest, most complete, and consistent for raising alcohol taxes (7). Meta-analyses find that higher alcohol taxes reduce excessive drinking as well as alcohol-related injuries, diseases, and death (8,9). Increasing alcohol taxes is recommended by the Community Preventive Services Task Force to reduce excessive alcohol use and related harms (10).

Although some states apply value-based alcohol taxes (ie, taxes based on a percentage of price) (11), all 50 states levy volume-based excise taxes (ie, taxes based on a fixed dollar amount per unit volume) as their principal form of taxation. Because excise taxes are not based on a percentage of the price of alcohol, inflation erodes their value unless they are increased. As of 2009, the average state beer tax had declined by approximately 70% in “real dollar” (ie, inflation-adjusted) terms since 1970, and the inflation-adjusted federal beer tax had declined by approximately 40% since its last increase in 1991 (12). In addition, current alcohol taxes do not cover alcohol-related costs. In 2006, the combined federal and state taxes on alcoholic beverages were approximately $0.12 per drink, while the median cost to state governments (in the form of health care expenditures, criminal justice system costs, etc.) was $0.78 per drink (13,14).

Despite their effectiveness and potential to generate government revenue to offset costs, increased taxes encounter robust political opposition. Principal concerns raised by opponents of alcohol tax increases are that 1) alcohol taxes are unfair to ”responsible” (ie, nonexcessive) drinkers; and 2) the cost of alcohol tax increases are disproportionately paid by those who are economically disadvantaged (15–17). Although it is true that alcohol taxes are not applied differentially based on a consumer’s drinking pattern or income, over 90% of adult excessive drinkers binge drink (consume ≥4 drinks per occasion for women, and ≥5 drinks per occasion for men), over half of the alcohol consumed by US adults is in the form of drinking binges, and the prevalence of alcohol consumption and binge drinking increases with household income (18,19). These facts suggest that the counterarguments may be invalid.

Because these counterarguments have not been empirically addressed at the state level, and because they are an important obstacle to maintaining or increasing state alcohol taxes, the objective of this study was to assess the impact of a series of hypothetical state alcohol tax increases on the total cost of alcohol (product plus tax) to adults in all 50 US states on the basis of alcohol consumption and sociodemographic characteristics. Information from this study is important for state policy makers and citizens and to inform evidence-based public health practice for treating a leading preventable cause of death in the United States.

## Methods

State-specific data on alcohol consumption were obtained from the 2011 Behavioral Risk Factor Surveillance System (BRFSS). A detailed overview of the BRFSS, including survey methods and information on data weighting, is available at www.cdc.gov/brfss/ and www.cdc.gov/brfss/technical_infodata/quality.htm. In brief, the BRFSS is a state-based random–digit-dial telephone survey of people aged 18 years or older that is conducted monthly in all states, the District of Columbia (DC), and 3 territories and coordinated by the Centers for Disease Control and Prevention (CDC). The BRFSS core alcohol questions (ie, questions asked of all drinkers in all states) were used to assess drinking frequency, average number of drinks consumed during drinking days, binge drinking frequency, and binge drinking intensity (ie, average largest number of drinks consumed on any occasion) within the past 30 days among US adults aged 18 or older. Weighted data from the 50 US states and Washington, DC, were used in this study.

Excessive drinking was defıned as binge drinking, heavy drinking, or any alcohol consumption by respondents aged 18 to 20 years of age (ie, adults under the minimum legal drinking age). Binge drinking was defined as consuming 5 or more drinks for men or 4 or more drinks for women on 1 occasion or more (20). Heavy drinking was defined as consuming an average of more than 2 drinks per day for men or more than 1 drink per day for women. Average daily alcohol consumption was calculated by multiplying the number of drinking days in the past 30 days by the usual number of drinks consumed during drinking days and then dividing by 30 (21). Nonexcessive drinkers were those who consumed alcohol in the past 30 days but were not classified as excessive drinkers.

The net cost of alcohol tax increases was calculated on the basis of change in the retail price of alcohol that would be expected to occur following a hypothetical tax increase. Because state-specific data about alcohol prices do not exist, we used national data from IMPACT Databank (22), which reports the average on- and off-premises price for beer, wine, and spirits, respectively. The average price of a standard drink in all 50 states and Washington, DC, was calculated by using beverage-specific price data from IMPACT Databank, weighted by state-specific data on beverage-specifıc per capita alcohol consumption using data from the Alcohol Epidemiologic Data System (http://www.healthindicators.gov/Resources/DataSources/AEDS_172/Profile). Our model assumed that 75% of the alcohol sold in the United States is purchased in off-premises establishments (eg, liquor stores, grocery stores, and other retail locations) (23–25). This assumption enabled us to calculate weighted cost per drink in each state.

The state-specific tax-per-drink was calculated by combining all state taxes (ie, excise, ad valorem, and sales taxes) for beer, wine, and spirits to determine a weighted average of state taxes paid per drink (26). State-specific tax rates for 2011 were obtained from the Alcohol Policy Information System, administered by the National Institute on Alcohol Abuse and Alcoholism (11). Among states with monopolies on wholesaling or retailing (or both) of spirits or wine (“control states”), there are no data about effective tax rates on monopolized beverages because states apply a series of markups on the retail price of the controlled beverage(s), and in some cases, the markup procedures are not transparent. However, in states with exclusively private retailing of spirits or wine (“noncontrol states”), there is a strong correlation between the taxes paid for a drink of beer and the average tax paid for a drink of any type (26). Using these correlation data and beer tax data from control states, we built a linear regression model to interpolate the average tax for monopolized beverages in control states.

The total cost per drink was calculated by summing the average price and average tax per drink in each state. Once the cost per drink was established, a revised cost per drink (product plus tax) was calculated for each of the series of hypothetical tax increases. The expected reduction in alcohol consumption following tax-related price increases was calculated by using beverage-specific estimates of price elasticity from a recent meta-analysis (8). Assuming a 100% pass-through of the tax to the price paid by consumers, we calculated the expected decreases in consumption for tax increases of $0.05, $0.10, and $0.25 per drink, and following a 5% increase in the retail price of a drink. After factoring in the impact of the price increase on average daily consumption, annual alcohol consumption was calculated by multiplying the average number of drinks per day by 365. The net cost of the hypothetical alcohol tax increase for individuals was then calculated by subtracting current annual expenditures from the total annual expenditures anticipated after each of the hypothetical tax increases. Per capita and aggregate net cost increases were then assessed among subgroups on the basis of alcohol consumption patterns (excessive, nonexcessive, nondrinkers), annual household income (<$25,000, $25,000–<$50,000, $50,000–<$75,000, ≥$75,000), employment status (employed for wages, nonemployed [ie, unemployed, self-employed, homemakers, students, people who are retired, and people who are unable to work]), and race/ethnicity (non-Hispanic white, other).

## Results

In 2011, 55.1% of US adults reported drinking in the past 30 days. Overall, 33.6% of US adults (60.9% of current drinkers) were classified as nonexcessive drinkers, and 21.5% of US adults (39.1% of current drinkers) were classified as excessive drinkers (Table 1). Table 1 also illustrates the characteristics of nonexcessive and excessive drinkers in terms of sociodemographic factors and the baseline number of drinks consumed annually among those groups. Among nonexcessive drinkers, for example, those with the highest incomes and non-Hispanic whites constituted the largest proportion of the population and also consumed more drinks annually on average.

**Table 1:** Population Distributions and Mean Number of Drinks Consumed at Baseline, by Drinking Status and Sociodemographic Characteristics, US Adults Aged ≥18 Years, Behavioral Risk Factor Surveillance System, 2011

Sociodemographic Characteristics	Nonexcessive Drinkers^a^ (n = 73,480,718)		Excessive Drinkers^b^ (n = 47,200,442)		Nondrinkers^c^ (n = 98,262,571)
	Population Proportion, %	Mean No. of Drinks Per Year	Population Proportion, %	Mean No. of Drinks Per Year	Population Proportion, %
**All respondents**	100	128.0	100	689.1	100
**Annual household income, $**					
<25,000	19.5	102.1	23.8	689.5	41.0
25,000–<50,000	25.3	124.6	25.1	722.1	27.3
50,000–<75,000	18.4	128.7	16.9	678.6	13.3
≥75,000	37.0	144.5	34.1	689.4	18.4
**Employment status^d^**					
Employed	57.3	125.9	66.5	696.3	44.6
Nonemployed	42.7	131.6	33.5	674.0	55.4
**Race/ethnicity**					
Non-Hispanic white	87.8	133.4	85.3	703.8	73.4
Other^e^	12.2	105.8	14.7	632.8	26.6

a Nonexcessive drinkers (33.6% of population, 60.9% of drinkers) were defined as respondents who consumed alcohol during the past 30 days but did not binge drink (≥5 drinks for men or ≥4 drinks for women on at least one occasion), was not categorized as a heavy drinker (daily average of >2 drinks for men or >1 drink for women [ie, >60 drinks per month for men or >30 drinks per month for women]), and aged 21 years or older.

b Excessive drinkers (21.5% of population, 39.1% of drinkers) were defined as respondents who during the past 30 days either engaged in binge drinking (≥5 drinks for men or ≥4 drinks for women on at least one occasion), was categorized as a heavy drinker (daily average of >2 drinks for men or >1 drink for women [ie, >60 drinks per month for men or >30 drinks per month for women]), or aged 18–20 years and reported drinking any alcohol.

c Nondrinkers were 44.9% of the population.

d Employment for wages was self-reported. Those who were nonemployed were unemployed, self-employed, homemakers, students, retired, or unable to work.

e “Other” racial/ethnic categories were non-Hispanic black, Hispanic white, Asian, or other race/ethnicity.

Among all adults in US states, excessive drinkers would pay about 5 times as much in additional net per capita costs (product plus tax) as nonexcessive drinkers for hypothetical alcohol tax increases, and nonexcessive drinkers would pay less than $10 annually for the largest ($0.25 per drink) tax increase (Table 2). For example, a $0.05 per drink tax increase would increase the state average annual cost of alcohol by $12.56 for excessive drinkers compared with $2.32 for nonexcessive drinkers, and a $0.25 per drink tax increase (the largest increase) would increase the average annual cost of alcohol by $49.49 for excessive drinkers compared with $9.15 for nonexcessive drinkers.

**Table 2:** Average Annual Per Capita Cost^a^ of 4 Alcohol Tax Increases, by Drinking Status, Income, Employment Status, and Race/Ethnicity, US Adults Aged ≥18 Years,^b^ Behavioral Risk Factor Surveillance System, 2011

Characteristics	Mean Annual Per Capita Net Cost in US $ (state minimum, state maximum)			
	$0.05 per drink increase	$0.10 per drink increase	$0.25 per drink increase	5% increase in drink price
**Alcohol consumption**				
Excessive^c^	12.56 (10.42–6.39)	23.79 (19.69–31.13)	49.49 (40.67–65.42)	19.38 (18.37–25.58)
Nonexcessive^d^	2.32 (1.98–2.96)	4.40 (3.75–5.64)	9.15 (7.7–12.00)	3.59 (2.97–4.91)
Nondrinker	NA	NA	NA	NA
**Household income, $**				
<25,000	2.95 (0.97–4.64)	5.59 (1.86–8.81)	11.64 (3.95–18.39)	4.55 (1.58–7.21)
25,000–<50,000	3.48 (2.19–4.69)	6.60 (4.17–8.89)	13.74 (8.78–18.48)	5.37 (3.11–6.80)
50,000–<75,000	3.60 (3.52–5.31)	6.83 (6.69–9.99)	14.19 (14.09–20.20)	5.55 (5.37–8.29)
≥75,000	4.31 (3.44–5.28)	8.16 (6.50–9.98)	16.98 (13.39–20.61)	6.68 (5.16–9.31)
**Employment status**				
Employed for wages^e^	4.10 (2.70–5.01)	7.77 (5.12–9.43)	16.15 (10.58–19.12)	6.33 (4.10–7.34)
Unemployed^f^	2.62 (1.15–4.17)	4.97 (2.20–7.91)	10.35 (4.66–16.51)	4.05 (1.86–6.48)
**Race/ethnicity**				
Non-Hispanic white	3.75 (2.50–4.37)	7.10 (4.73–8.55)	14.75 (9.83–17.77)	5.77 (3.8–6.86)
Other^g^	2.77 (1.97–3.33)	5.26 (3.75–6.33)	10.96 (7.82–13.20)	4.31 (3.07–5.19)

Abbreviation: NA, not applicable.

a The net cost is the change in the amount a person would spend on alcohol (product plus tax) as a result of a hypothetical tax increase. This table includes all US adults, so average per capita costs are calculated by dividing costs among drinkers and nondrinkers in each group.

b All US adults, including nondrinkers.

c An excessive drinker was defined as a respondent who during the past 30 days either engaged in binge drinking (≥5 drinks for men or ≥4 drinks for women on at least one occasion), was categorized as a heavy drinker (daily average of >2 drinks for men or >1 drink for women, [ie, >60 drinks per month for men or >30 drinks per month for women]), or who was aged 18–20 years and reported drinking any alcohol.

d A nonexcessive drinker was defined as a respondent who consumed alcohol during the past 30 days but did not binge drink (≥5 drinks for men or ≥4 drinks for women on at least one occasion), was not categorized as a heavy drinker (daily average of >2 drinks for men or >1 drinks for women, [ie, >60 drinks per month for men or >30 drinks per month for women]), and who was aged 21 years or older.

e Self-reported.

f Self-reported; includes self-employed, unemployed, homemaker, student, retired, or unable to work.

g Other race/ethnicity includes non-Hispanic black, Hispanic white, Asian, and other racial/ethnic group.

By sociodemographic characteristics, average per capita costs among US adults (including nondrinkers) from tax increases would be higher among people with higher incomes, employed people, and non-Hispanic whites (Table 2). For example, after a tax increase of $0.25 per drink, people earning less than $25,000 would pay an average additional cost of $11.64 per year, while those earning $75,000 or more would pay an additional $16.98 per year. Similarly, following this same hypothetical tax increase, those who are employed would pay more on average than nonemployed people ($16.15 vs $10.35 annually), and non-Hispanic whites would pay more on average than people of other racial/ethnic groups ($14.75 vs $10.96).

Among current drinkers, stratified by drinking group and sociodemographic characteristics (Table 3), nonexcessive drinkers would pay little in increased per capita costs; for example, the highest average per capita annual cost in any stratum of income, employment, and racial/ethnicity was $10.31 for the largest tax increase ($0.25 per drink). In addition, excessive drinkers would pay 4.8 to 6.8 times more in per capita average annual costs than would nonexcessive drinkers. In terms of aggregate costs, all strata of nonexcessive drinkers would pay less than 30% of costs compared with their corresponding groups of excessive drinkers, although there are more nonexcessive drinkers than excessive drinkers.

**Table 3:** Average Annual Per Capita Net Cost^a^ for Nonexcessive Drinkers,^b^ by Sociodemographic Factors, Excessive^c^ Drinker: Nonexcessive Drinker Per Capita Cost Ratio, and Proportion of Total Costs Paid by Nonexcessive Drinkers, US Adults Aged ≥18 Years, Behavioral Risk Factor Surveillance System, 2011

Sociodemographic characteristics	Mean Annual Per Capita Net Cost From Alcohol Tax Increases for Nonexcessive Drinkers (State Minimum, State Maximum)				Excessive Drinker: Nonexcessive Drinker Per Capita Cost Ratio (State Range)	Percentage of Aggregate Costs Paid by Nonexcessive Drinkers (State Range)
	$0.05 Per Drink Increase	$0.10 Per Drink Increase	$0.25 Per Drink Increase	5% Increase in Drink Price		
**Annual household income, $**						
<25,000	1.86 (1.37–2.51)	3.52 (2.57–4.76)	7.33 (5.14–10.02)	2.87 (2.01–3.94)	6.8 (5.1–8.2)	13.8 (11.2–16.3)
25,000–<50,000	2.26 (1.70–3.36)	4.28 (3.23–6.40)	8.90 (6.74–13.55)	3.49 (2.60–5.43)	5.8 (4.4–7.0)	21.1 (15.2–25.2)
50,000–<75,000	2.33 (1.83–2.98)	4.43 (3.47–5.68)	9.20 (7.23–12.08)	3.61 (2.79–4.94)	5.3 (4.1–6.7)	26.2 (21.2–30.1)
≥75,000	2.61 (2.13–3.46)	4.96 (4.01–6.60)	10.31 (8.22–14.03)	4.06 (3.09–5.74)	4.8 (3.9–6.4)	28.5 (23.4–34.2)
**Employment status**						
Employed^d^	2.28 (1.96–2.83)	4.33 (3.70–5.39)	9.00 (7.63–11.46)	3.53 (2.92–4.69)	5.6 (4.5–7.1)	21.2 (17.2–24.5)
Nonemployed^e^	2.39 (2.00–3.28)	4.53 (3.78–6.25)	9.42 (7.78–13.28)	3.70 (2.88–5.43)	5.1 (4.1–6.4)	25.0 (21.2–29.4)
**Race/ethnicity**						
White, non-Hispanic	2.48 (2.01–3.32)	4.70 (3.85–6.34)	9.76 (7.94–13.35)	3.83 (2.97–5.57)	5.4 (4.2–6.7)	23.8 (20.1–26.3)
Other^f^	1.88 (1.38–2.53)	3.57 (2.63–4.88)	7.44 (5.22–10.09)	2.93 (2.09–4.02)	5.9 (4.5–7.0)	18.8 (16.7–22.1)

a The net cost is the change in the amount of money a person would spend on alcohol (product plus tax) as a result of hypothetical tax increases.

b A nonexcessive drinker is defined as a respondent who consumed alcohol during the past 30 days but within those 30 days did not binge drink (≥5 drinks for men or ≥4 drinks for women on at least one occasion), was not categorized as a heavy drinker (daily average of >2 drinks for men or >1 drink for women [ie, >60 drinks per month for men or >30 drinks per month for women]), and who was aged 21 or older.

c An excessive drinker is defined as respondent who during the past 30 days either engaged in binge drinking (≥5 drinks for men or ≥4 drinks for women during at least one occasion) or was categorized as a heavy drinker (daily average of >2 drinks for men or >1 drink for women [ie, >60 drinks per month for men or >30 drinks per month for women]), or who was aged 18–20 and reported drinking any alcohol.

d Self-reported.

e Nonemployed includes self-employed, unemployed, homemaker, student, retired, or unable to work.

f Other race/ethnicity includes non-Hispanic black, Hispanic white, Asian, and other racial/ethnic group.

By income, per capita costs were lower for those with less income, and the percentage of aggregate costs paid by nonexcessive drinkers as a group was lower among those earning less income (eg, those earning <$25,000 would pay 13.8% of the aggregate costs, while those earning $75,000 or more annually would pay 28.5% of costs) (Table 3). Among nonexcessive drinkers, the average annual per capita cost of tax increases was similar by employment status, but in aggregate, the nonemployed paid only 25.0% of costs. People from racial/ethnic minorities would also pay less in annual per capita costs and a lower proportion of aggregate costs (18.8%, state range 16.7%–22.1%) than would non-Hispanic whites (23.8%, range 20.1%–26.3%). Additional state-specific estimates of the costs from each of the 4 hypothetical tax increases for nonexcessive and excessive drinkers are available at http://www.camy.org/action/taxes/taxtool/index.html.

By state, the average annual increase in per capita costs to nonexcessive drinkers from even the smallest tax increase ($0.05 per drink) did not exceed $3.00 (Table 4). Similarly, the increase in per capita costs from the largest tax increase ($0.25 per drink) did not exceed $12.00 in any state. In contrast, excessive drinkers would pay from 4.1 (District of Columbia and Tennessee) to 7.6 (Arkansas) times more in average annual per capita costs than nonexcessive drinkers. Nonexcessive drinkers in Indiana had the lowest average annual increase in costs, while those in Maine had the highest average annual increase in costs. The proportion of total costs paid by nonexcessive drinkers as a group ranged from 16.5% (Hawaii, Utah) to 31.3% (Tennessee).

**Table 4:** State-Specific Average Annual Per Capita Net Cost^a^ for Nonexcessive Drinkers^b^, Excessive Drinker^c^: Nonexcessive Drinker Per Capita Cost Ratio, and Proportion of Total Costs Paid by Nonexcessive Drinkers, US Adults Aged ≥18 Years, Behavioral Risk Factor Surveillance System, 2011

State	Mean Annual Per Capita Net Cost ($) From Tax Increases for Nonexcessive Drinkers				Excessive Drinker: Nonexcessive Drinker Per Capita Cost Ratio	Percentage of Aggregate Costs Paid by Nonexcessive Drinkers
	$0.05 Per Drink	$0.10 Per Drink	$0.25 Per Drink	5% of Drink Price		
Alabama	2.39	4.54	9.56	3.65	5.8	19.8
Alaska	2.44	4.61	9.39	3.77	5.5	21.7
Arizona	2.36	4.47	9.27	3.61	4.7	24.6
Arkansas	2.15	4.08	8.58	3.36	7.6	17.0
California	2.22	4.22	8.81	3.59	5.2	22.8
Colorado	2.38	4.47	9.06	3.52	4.6	27.9
Connecticut	2.34	4.41	9.06	3.79	4.8	29.1
Delaware	2.20	4.13	8.25	3.23	5.6	20.3
District of Columbia	2.53	4.78	9.86	4.45	4.1	24.3
Florida	2.31	4.36	9.03	3.65	5.6	23.9
Georgia	2.32	4.39	9.10	3.48	6.0	20.7
Hawaii	2.44	4.64	9.70	3.83	5.8	16.5
Idaho	2.35	4.46	9.37	3.90	5.2	23.4
Illinois	2.22	4.21	8.79	3.45	6.2	17.7
Indiana	1.98	3.75	7.71	2.97	5.8	20.9
Iowa	2.22	4.21	8.75	3.22	6.3	18.1
Kansas	2.25	4.26	8.89	3.40	5.6	22.7
Kentucky	2.12	4.03	8.40	3.25	6.4	16.7
Louisiana	2.16	4.09	8.42	3.12	6.4	18.3
Maine	2.68	5.08	10.53	4.15	4.9	27.8
Maryland	2.18	4.11	8.34	3.34	5.2	25.2
Massachusetts	2.55	4.82	9.89	3.99	4.8	26.6
Michigan	2.27	4.28	8.79	3.43	5.6	21.1
Minnesota	2.28	4.30	8.83	3.59	5.2	22.1
Mississippi	2.15	4.08	8.51	3.15	6.2	19.3
Missouri	2.16	4.08	8.36	3.14	6.0	18.8
Montana	2.63	4.97	10.21	3.73	5.0	22.5
Nebraska	2.26	4.29	8.95	3.27	5.6	19.9
Nevada	2.33	4.41	9.11	3.62	5.7	22.0
New Hampshire	2.57	4.83	9.76	4.00	5.4	26.8
New Jersey	2.17	4.10	8.43	3.53	5.2	26.8
New Mexico	2.49	4.73	9.85	3.74	5.2	24.9
New York	2.27	4.29	8.83	3.53	4.9	25.1
North Carolina	2.55	4.84	10.16	3.97	5.1	23.7
North Dakota	2.05	3.86	7.89	3.01	5.4	19.9
Ohio	2.38	4.53	9.57	3.50	5.7	20.3
Oklahoma	2.02	3.84	8.11	3.14	6.4	17.9
Oregon	2.48	4.67	9.54	3.59	5.1	27.7
Pennsylvania	2.39	4.53	9.47	3.47	5.4	23.6
Rhode Island	2.33	4.41	9.07	3.70	4.6	26.2
South Carolina	2.42	4.59	9.58	3.67	5.9	20.8
South Dakota	2.19	4.14	8.52	3.15	5.1	21.5
Tennessee	2.70	5.15	10.91	4.37	4.1	31.3
Texas	2.38	4.53	9.67	3.75	5.9	20.0
Utah	2.33	4.40	9.10	3.53	5.7	16.5
Vermont	2.96	5.64	12.00	4.91	4.5	30.1
Virginia	2.64	5.00	10.47	4.06	5.0	25.7
Washington	2.58	4.89	10.25	4.20	4.3	29.5
West Virginia	2.38	4.52	9.51	3.37	6.8	19.7
Wisconsin	2.35	4.42	8.97	3.44	5.1	20.9
Wyoming	2.37	4.45	8.96	3.37	5.7	20.8

a The net cost is defined as the change in the amount of money a person would spend on alcohol (product plus tax) as a result of hypothetical tax increases.

b A nonexcessive drinker was defined as a respondent who consumed alcohol during the past 30 days but within those 30 days did not binge drink (≥5 drinks for men or ≥4 drinks for women on at least one occasion), was not categorized as a heavy drinker (daily average of ≥2 drinks for men or ≥1 drink for women [ie, ≥60 drinks per month for men or ≥30 drinks per month for women]), and who was 21 years of age or older.

c An excessive drinker is defined as a respondent who during the past 30 days either engaged in binge drinking (≥5 drinks for men or ≥4 drinks for women during at least one occasion) or was categorized as a heavy drinker (daily average of ≥2 drinks for men or ≥1 drinks for women [ie, ≥60 drinks per month for men or ≥30 drinks per month for women]), or who was 18 to 20 years of age and reported any alcohol consumption.

## Discussion

On a per capita basis, nonexcessive drinkers would typically pay less than $10.00 annually in average net costs for the largest hypothetical state alcohol tax increase ($0.25 per drink) and less than $2.50 annually for the smallest tax increase ($0.05 per drink). Furthermore, excessive drinkers would pay more than 5 times as much as nonexcessive drinkers in per capita increased costs. As a group, excessive drinkers would also pay approximately three-quarters of the aggregate costs, even though nonexcessive drinkers outnumber excessive drinkers.

Because excessive drinkers account for most alcohol-related harms in the United States (5), their increased cost relative to nonexcessive drinkers appears justifiable. That excessive drinkers would pay most of the increased cost for state alcohol tax increases is not surprising and reflects the skewed distribution of alcohol consumption in the United States. More than half of the alcohol consumed by US adults is in the form of binge drinks, and binge drinkers were responsible for about three-quarters of the $249 billion in economic costs due to excessive drinking in the United States in 2010 (5).

Members of low-income households and racial/ethnic minorities, including those who are nonexcessive drinkers, would also pay less in per capita and aggregate costs following tax increases compared with non-Hispanic whites or those with higher incomes. These findings contradict the belief that those who are economically disadvantaged would bear most of the costs from alcohol tax increases.

The finding that the cost of state alcohol tax increases would be similar for excessive drinkers in lower and higher income groups is consistent with the results of other studies (27). However, increasing the price of alcohol might be a particularly effective way to reduce the risk of alcohol-related harms in low-income populations. Binge drinking intensity (the number of drinks consumed during a binge drinking episode) is higher among low-income groups compared with high-income groups (19), and proportionate reductions in consumption among those consuming on the “steep” portion of the risk curve would yield larger reductions in risk relative to those who drink less.

This research has limitations. First, because there are no adequate state-specific estimates of alcohol prices in the United States, national estimates of prices were added to state-specific taxes to estimate state prices for alcohol. Therefore, we may have overestimated the increase in net costs to drinkers in states with alcohol prices that are below the national estimates, because these states would have both a lower pre-tax price on alcohol and experienced a greater reduction in consumption due to higher percentage increases in post-tax retail prices. Second, we used beverage-specific price elasticities from a meta-analysis (8). Price elasticities may vary on the basis of many factors (age, income, drinking pattern); however, across the literature, estimates are inconsistent and there has been no systematic review or meta-analysis of tax elasticities on the basis of these factors. Were the price elasticity for alcohol more negative for nonexcessive drinkers compared with excessive drinkers, excessive drinkers would have paid more in per capita and aggregate costs from alcohol tax increases because their drinking would have been relatively less affected by price increases. Similarly, were the price elasticity more negative for low-income persons, high-income persons would have paid relatively more in cost increases. Third, it is possible that those who drink more may purchase cheaper alcohol, so that a given excise tax increase may represent a greater percentage increase in cost and a larger decrease in consumption compared with those who drink less when price elasticity is applied (6).

Surveys based on self-report are subject to nonresponse bias, and alcohol consumption may be underreported among respondents (28). Therefore, our cost estimates are likely to be conservative, particularly for excessive drinkers who are particularly prone to underreporting their consumption and who may also be less likely to participate in surveys or other alcohol studies (29,30). Because this was a study of the change in the total cost of alcohol (product plus tax) due to tax increases, it is important to note that total cost increases are less than the amount paid in additional taxes because total costs are offset by the fact that consumers would purchase less alcohol in response to increased prices. However, reporting net cost is preferable because it represents the “bottom line” in terms of how much more consumers would pay for alcohol from tax increases.

Although many evidence-based strategies exist for reducing excessive alcohol use, a recent study found that a few policies that affect the price and availability of alcoholic beverages, including higher alcohol taxes and controls on alcohol outlet density, have the greatest impact on binge drinking among US adults (31). Raising alcohol taxes could also reduce the gap between alcohol-related costs incurred by state governments versus the revenues derived from state alcohol taxes.

State-specific tax increases would cost more for excessive drinkers, those with higher incomes, and non-Hispanic whites. Costs to nonexcessive drinkers would be modest. This study may be helpful in educating policy makers and the public about the distribution of costs from state alcohol tax increases and can inform debate about the promulgation of evidence-based public health practices to reduce a leading preventable cause of death and social problems in the United States.
